# Tunable Multiple-Step Plasmonic Bragg Reflectors with Graphene-Based Modulated Grating

**DOI:** 10.3390/s16122039

**Published:** 2016-12-01

**Authors:** Qinglu Qian, Youjian Liang, Yue Liang, Hongyan Shao, Menglai Zhang, Ting Xiao, Jicheng Wang

**Affiliations:** 1 School of Science, Jiangsu Provincial Research Center of Light Industrial Optoelectronic Engineering and Technology, Jiangnan University, Wuxi 214122, China; qianqinglu0915@163.com (Q.Q.); liangyoujian327@163.com (Y.L.); liangyue1012@163.com (Y.L.); shaohongyan_1992@163.com (H.S.); 15061885471@163.com (M.Z.); xiaoting_0130@163.com (T.X.); 2 Key Laboratory of Semiconductor Materials Science, Institute of Semiconductors, Chinese Academy of Sciences, P.O. Box 912, Beijing 100083, China

**Keywords:** plasmonics, Bragg grating sensor, metal-insulator-metal, finite element method

## Abstract

We propose a novel plasmonic Bragg reflector (PBR) based on graphene with multiple-step silicon structure. The monolayer graphene bears locally variable optical properties by modulation of electric fields, and the periodical change of effective refractive index on graphene can be obtained by external bias voltage in the mid-infrared region. Through patterning the PBR units into multiple-step structures, we can decrease the insertion loss and suppress the rippling in transmission spectra. By introducing the defect into the multiple-step PBRs, the multiple resonance modes are formed inside the stopband by increasing the step number. This work may pave the ways for the further development of ultra-compact low-cost hyperspectral sensors in the mid-infrared region.

## 1. Introduction

Recently, graphene has become a popular research field and has attracted an increasing amount of attention [1]. Graphene, as a monolayer of carbon atoms arranged in a honeycomb lattice, offers a promising platform to overcome these obstacles [2,3]. Due to the unique electronic structure and dynamic tunability, graphene bears stronger mode confinement and lower propagation losses, and can be tuned by applying external gate voltages [4,5]. Graphene plasmonics, similar to metal plasmonics at the visible region, can be easily induced in the near-infrared to terahertz (THz) regime. In particular, the surface charge density, namely the chemical potential, can be actively modified via chemical doping or with the external gate voltage, thus giving rise to dramatic changes in the optical properties [6]. Surface plasmon polaritons (SPPs) bound to graphene display a strong field confinement, already verified by experiments [7,8]. These remarkable and outstanding properties enable a utility optical material in optoelectronic applications. Consequently, more attention has been focused on graphene-based plasmonic waveguides [9,10,11,12,13,14,15,16,17] in recent years. In addition, the tunable nano-modulators based on graphene plasmonic waveguide modulators have been proposed and numerically demonstrated [18]. Lu et al. have designed a slow-light waveguide based on graphene and silicon graded grating [19]. The tunable plasmonic Bragg reflectors based on graphene silicon waveguide have been presented and studied [20].

In this paper, the plasmonic Bragg reflector (PBR) structure based on graphene with multiple-step units are numerically presented in the mid-infrared region. External bias voltages are applied to control the optical properties of the graphene, and the periodical modulation of effective refractive index on graphene is obtained to form a forbidden band on transmitted spectrum. The insertion loss is lower and the rippling in transmission is suppressed by increasing the step number of multiple-step structures. Furthermore, multiple peaks appear in the stopband while the defect introduces into the multiple-step PBRs. The finite element method (FEM) has been utilized to perform simulation work.

## 2. Model and Theoretical Analysis

**Figure 1:**
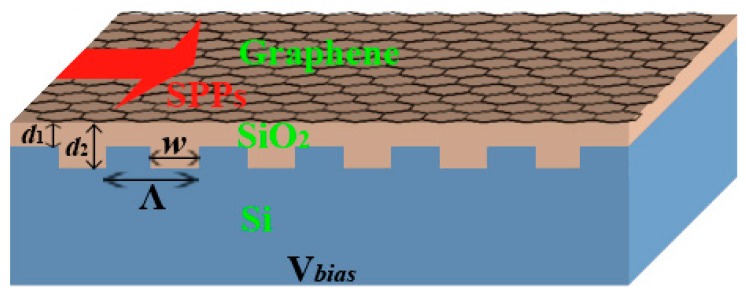
Schematic of the structure of a plasmonic Bragg reflector and the structure parameters are *w* = 40 nm and Ʌ = 80 nm. Voltage *V*_bias_ is applied between graphene and silicon grating.

The schematic of the PBR structure we proposed is shown in Figure 1. It consists of a monolayer graphene and a finite array of periodic highly doped silicon-based grating, and they are separated by a thin layer of silica. When a biased voltage *V_bias_* is applied between graphene and silicon grating, the periodic induced electric field can be obtained and modulated in graphene due to the periodic grating structure. This theoretic structure is not hard to build up experimentally. The grating silica/silicon substrate can be fabricated via electron beam lithography. The monolayer graphene can be prepared by the chemical vapor deposition method and then placed on the grating substrate. When a broadband mid-infrared irradiation is incident on the structure from the left interface, SPPs will be excited and propagate along the graphene layer. According the Bragg condition, only a narrowband of the spectrum can pass through the graphene waveguide and the rest will be reflected away., We set *d*(*y*), i.e., *d*_1_ and *d*_2_ labeled in Figure 1, as the thickness of the SiO_2_ thin layer, which is a periodic step function of *y*, and it is far less than the width of the trench in the grating. We assume the field is given by the equation *E*(*y*) = *ε*_SiO2_*V*/*d*(*y*); here, *V* = *V_bias_*, and *ε*_SiO2_ is the relative permittivity of SiO_2_. Then, the surface charge density of graphene can be approximated by *n*(*y*) = [*ε*_0_*E*(*y*)]/*e* = *ε*_0_*ε*_SiO2_*V*/*ed*(*y*); here, *ε*_0_ is the vacuum permittivity, and *e* is the elementary charge. Further, we can modulate the Fermi level according to *E_f_*(*y*) = *ħν_f_*(*πn_s_*)^1/2^; here, *ν_f_* = 10^6^ m/s is the Fermi velocity of the electron in the graphene, and *ħ* is the reduced Planck constant. The Fermi level affects the scattering of electrons, characterized by the relaxation time *τ* = *μE_f_*(*y*)/*eν_f_*^2^; here, *μ* is the carrier mobility. According to Kubo formula, the optical properties of a monolayer graphene can be characterized by a complex surface conductivity *σ_G_* [11]:

$$\begin{array}{cc} \sigma_{G}(y)= & \frac{2ie^{2}k_{B}T}{\pi \hbar^{2}[\omega +i\tau {\left(y\right)}^{-1}]}ln\left[2cosh[\frac{E_{f}(y)}{2k_{B}T}]\right]\\ & +\frac{ie^{2}}{4\pi \hbar }ln\left[\frac{2E_{f}(y)-\hbar [\omega +i\tau {\left(y\right)}^{-1}]}{2E_{f}(y)+\hbar [\omega +i\tau {\left(y\right)}^{-1}]}\right] \end{array}$$

The first term and the second term correspond to intraband transitions and interband transitions, respectively, where *k_B_* is the Boltzmann constant, *T* is the temperature, and w is the angular frequency of optical excitation. 

In our work, the permittivity of graphene layer [21,22,23] can be characterized by a dielectric function:

(1) $$\varepsilon_{G}=\varepsilon_{b}+\frac{i\sigma_{G}}{\varepsilon_{0}\omega t_{G}}$$

where *ε_b_* and *t_G_* are typically chosen as 2.5 and 1 nm, respectively. The optical parameters of other involved materials are *ε*_Air_ = 1, *ε*_SiO2_ = 3.9, and *ε*_Si_ = 11.7 in the spectral region of interest. In our study, the values of voltages and the parameters of the structure are dependent on each other and should be reasonably chosen.

We regard the structure as an Air-Graphene-SiO_2_-Si multi-layer structure. According to Maxwell’s equations and the boundary conditions, the general dispersion relation for the graphene waveguide in the multilayer structures can be solved by the following equation:

(2) $$\frac{\varepsilon_{1}}{\sqrt{N_{eff}^{2}-\varepsilon_{1}}}+\frac{\varepsilon_{2}}{\sqrt{N_{eff}^{2}-\varepsilon_{2}}}=-i\sigma_{G}\eta_{0}$$

where *η*_0_ is the intrinsic impedance of free space. If *ε*_1_ = *ε*_2_ *= ε*, the effective refractive can be reduced from Equation (3) as follows:

(3) Neff=Re(1−(2σGη0)2)

**Figure 2:**
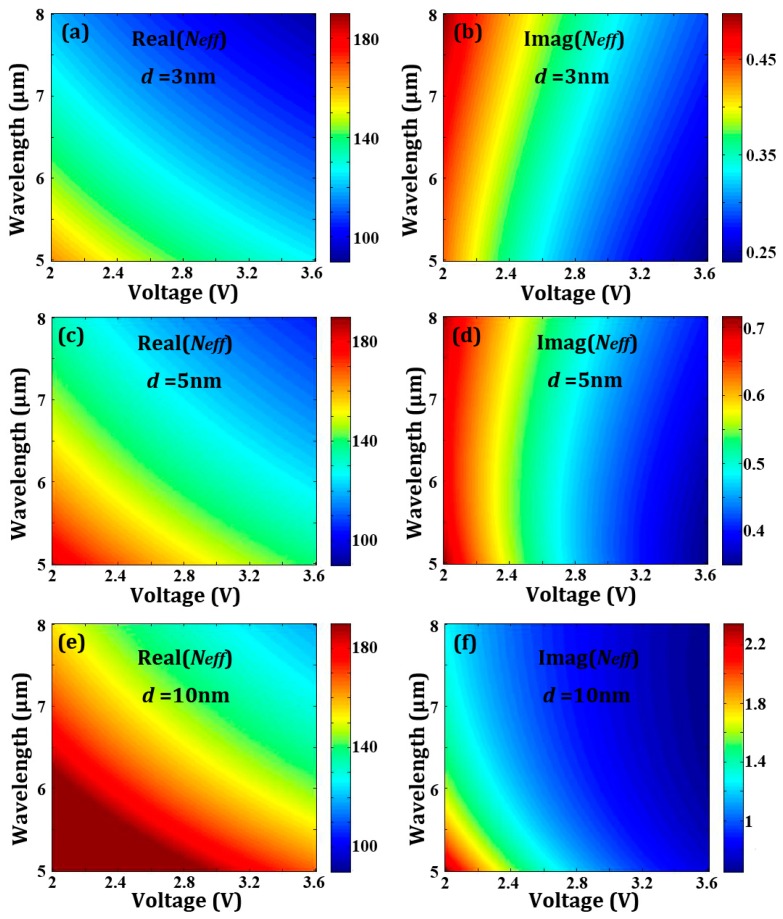
(**a**,**c**,**e**) The real part and (**b**,**d**,**f**) the imaginary part of the effective reflective index of graphene Neff as a function of applied voltage *V* and wavelength *λ* for *d* = 3 nm, *d* = 5 nm, and *d* = 10 nm. Here, the carrier mobility is *μ* = 2 m^2^·V^−1^·s^−1^.

The relation of the effective refractive *N_eff_* (both real and imaginary parts) with bias voltage *V* and wavelength *λ* is well depicted in Figure 2a–d. Here, the carrier mobility is *μ* = 2 m^2^·V^−1^·s^−1^, and the real parts are invariant while the imaginary parts decrease dramatically as *μ* increases.

## 3. Simulations and Results

**Figure 3:**
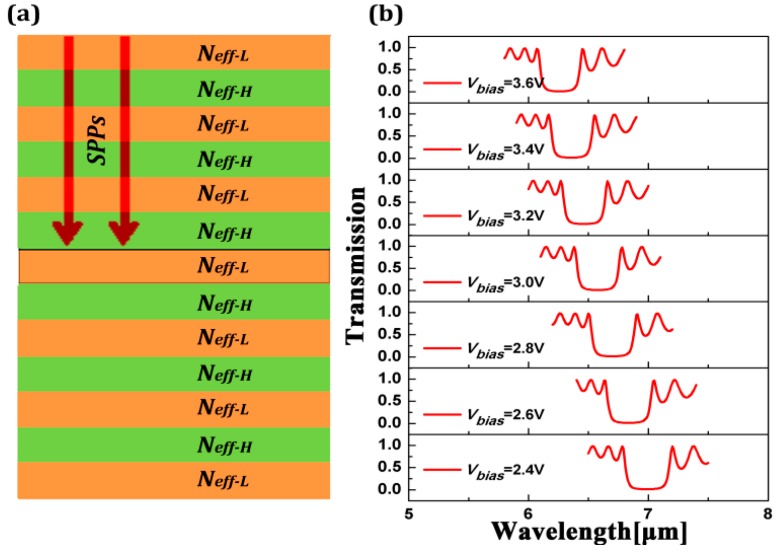
(**a**) The principle model of the graphene waveguide, and *N_eff-L_* and *N_eff-H_* are the effective reflective index of graphene at sections of *d*_1_ = 5 nm and *d*_2_ = 10 nm, respectively; (**b**) The position of the stop-band in the transmission spectrum can be controlled by changing the bias voltage *V_bias_*.

The loss of the graphene-based structure is mainly caused by the imaginary part of complex permittivity of graphene. Here, we firstly consider the real part of complex permittivity of graphene and its lossless style. When SPPs propagate in periodically modulating media, we can regard the graphene-based silica grating structure as a Bragg reflector made of alternating layers with a high reflective index (*N_eff-H_*) and a low reflective index (*N_eff-L_*), as shown in Figure 3a. The partial reflection will happen in each boundary due to the mismatch between the adjacent effective refractive index. High reflection will occur if the reflected beams superpose constructively and a stop-band will appear in the transmission spectrum, as shown in Figure 3b. We can see that the position of the stop-band is blue-shifted as *V_bias_* increases. Here, the parameters are set as *d*_1_ = 5 nm, *d*_2_ = 10 nm, *w* = 40 nm, and Ʌ = 80 nm.

**Figure 4:**
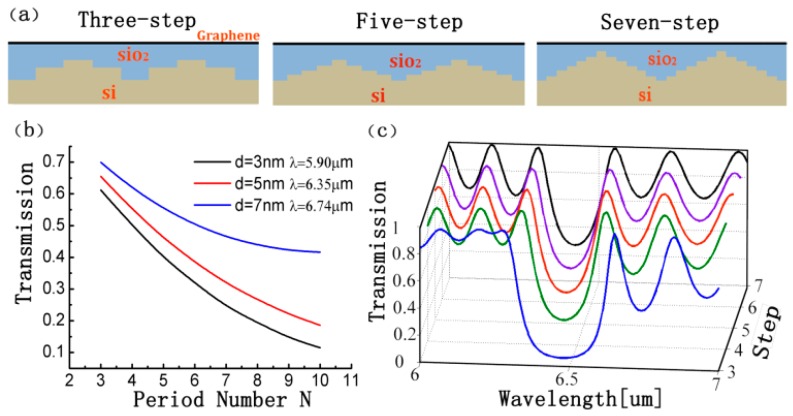
(**a**) The 2D schematic illustration of the PBR unit designed into multiple-step patterns: three-step PBR unit, five-step PRB unit, and seven-step PBR unit, respectively; (**b**) The transmission of the three-step PBR with a different period number *N*; (**c**) The transmission of the PBRs for different cases from the three-step to seven-step pattern structures, respectively. Here, *d* is set as 5 nm, *w* = 20 nm, the period number *N* = 6, and the bias voltages *V_bias_* = 3.2 V.

Furthermore, we design the PBR units into the multi-step pattern to deal with the high insertion loss and severe rippling in the transmission spectra resulting from the abrupt change of *N_eff_* in the groove depth [24,25]. The 2D schematic of the three-step, five-step, and seven-step versions of the PBR units are illustrated in Figure 4a. For the three-step PBR unit, we plot the transmission spectra in Figure 4b versus the period number *N* changing with deferent SiO_2_ thickness *d* and wavelength *λ*. After comparing the transmission spectra in Figure 4c, we find the expected enhancement on transmission spectra and rippling suppression. Additionally, the stopband is gradually narrowed when the PBR unit is changed into more steps. The multiple steps in a PBR unit actually adding multiple reflections in the Bragg reflections process make it harder to satisfy the Bragg conditions, which results in sidelobe suppression and a narrowed stopband.

**Figure 5:**
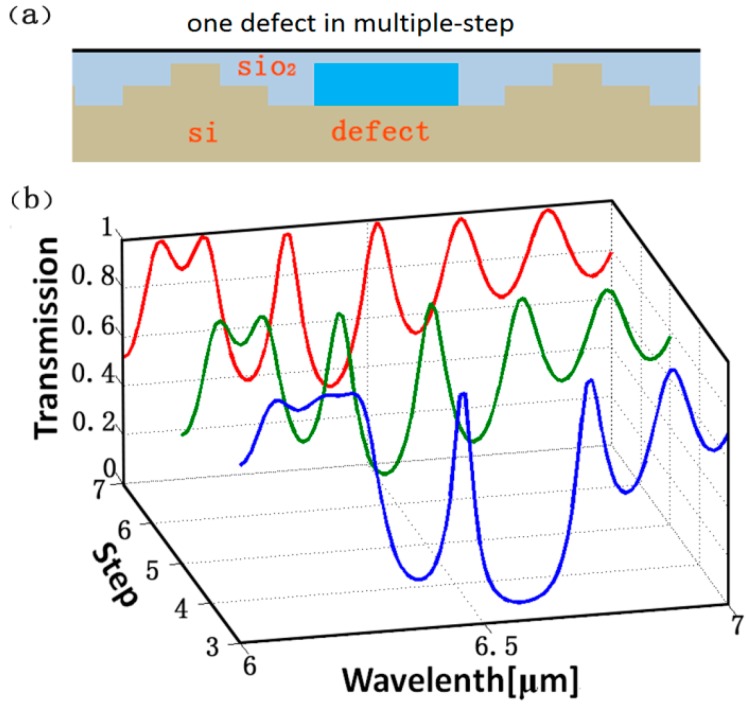
(**a**) Schematic illustration of 2D profiles of multiple-step structure with one defect; (**b**) The transmission of PBRs with the defect for different cases from the three-step to seven-step pattern structures, respectively. The parameters of the structures and the bias voltages are the same as those in Figure 4a. Here, the width of the defect t = 100 nm, and the gate voltage *V_g_* = 2.2 V.

In order to make the practical application of this ideal platform more extensive, we designed one defect structure in the multiple-step PBR reflectors to realize the filtering, as shown in Figure 5a. The parameters of the structures and the gate voltage are the same as those in Figure 4a, where the width of the defect *t* = 100 nm, and the gate voltage *V_g_* = 2.2 V. From Figure 5b, we can see that one peak appears in the stopband of the transmission spectrum for the three-step structure with the defect and two peaks for the five-defect and seven-defect structures, and that their resonance wavelengths of the projection have a slight red-shift and the transmissions of the peak are almost invariant when increasing the step number. The relative wide bandgaps near those peaks always exist. Those sharp projections, obtained by the hybrid effects of the first order mode resonance and high order mode resonance, can be regularly adjusted in a reasonable wavelength range.

**Figure 6:**
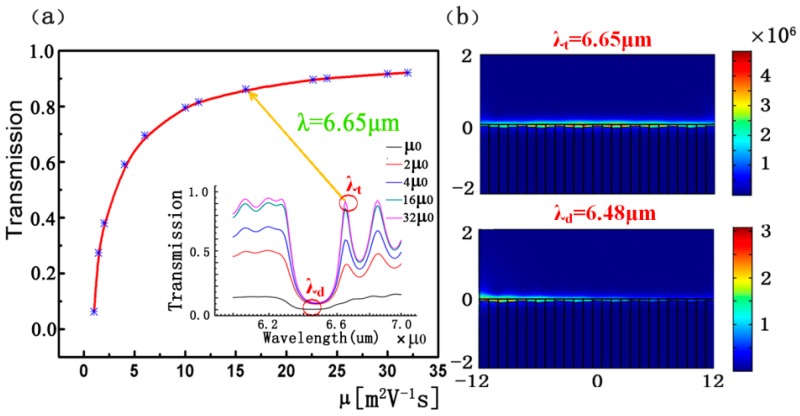
(**a**) The transmission at *λ_t_* = 6.65 μm as a function of carrier mobility for the three-step structure considering the material losses of graphene. The inset is the transmission spectrum for a different carrier mobility *μ*. Here, *μ*_0_ is set as 1 m^2^·V^−1^·s^−1^, and the bias voltages are *V_bias_* = 3.2 V; (**b**) The 2D distributions of the electric field |*E*|^2^ at excitation wavelengths *λ_t_* and *λ_r_* when *V_bias_* = 3.2 V.

Finally, we need to consider the material losses of graphene. As shown in Figure 6a, using the three-step structure as an example, the value of the transmission peak will increase with the increasing of carrier mobilities. In other words, the carrier mobility exerts an effect on the imaginary part of the effective refractive *N_eff_* of graphene. This corresponds to the preceding statement. Another conspicuous feature is that the effect decreases with the increase in carrier mobility when it exceeds 20 m^2^·V^−1^·s^−1^. Thus, we can consider the losses negligible in this case, and it can be realized by using proper doping or compensating the gain media. In Figure 6b, we plot the distributions of the electric field at excitation wavelengths *λ_t_* and *λ_r_* in the case of *V_bias_* = 3.2 V in order to visualize the propagating principle of the excited SPPs.

## 4. Conclusions

We have here presented some plasmonic Bragg reflectors based on graphene to realize the tunable low-loss filtering effect in the broadband multiple-step structures, and PBRs feasibilities have been adequately verified by FEM. By modulating the bias voltages of graphene, we can turn the transmission spectrum according to our need. Moreover, the transmission can reach above 80%, which means the losses are quite low as long as the carrier mobility is large enough. In addition, by designing the PRB units into multi-step structures, we can mitigate the insertion loss and suppress the rippling in transmission spectra. We introduced the defect structure in the multiple-step PBR reflectors to realize multiple filtering phenomena. These proposed designs were easily carried out in experiment. We hope they can help pave new ways in actively tunable sensoring applications.
